# Supplementary material for: How to choose a design thinking method for teaching the design of localization: A two-dimension linguistic fuzzy model with two-tuples
Source: PLoS One. 2024 May 30;19(5):e0300505. doi: 10.1371/journal.pone.0300505 (PMC11139305; doi:10.1371/journal.pone.0300505)
Supplement: S1 Fig — (DOCX) [file pone.0300505.s001.docx]

**S1 Fig. 22 categories of design thinking methods.**

| **NO.** | **Design Thinking Categories** | **Method of representation** |
| --- | --- | --- |
| **1** | Experimental testing | Experimental methods, usability testing, A/B testing, empirical design, ergonomic analysis |
| **2** | Property Analysis | AEIOU, Kano model, Component analysis, Elito |
| **3** | Feature categories | Card Sorting, KJ Method, Affinity Charts, Chakra Design, Chakra Visits |
| **4** | Internet Survey | Interference-free weblog research, automated remote research, moderate remote research |
| **5** | Divergent thinking | Brainstorming Image Organization Act, Thinking Maps |
| **6** | Information Analysis | Literature review, sub-studies |
| **7** | Content Search | Content analysis, content inventory and content review, task analysis |
| **8** | Humanities Exploration | Cultural Exploration, Design Ethnography, Diary Research, Guided Narrative |
| **9** | User Experience | UX Audit, UX Journey Mapping, Critical Incident Approach |
| **10** | Group Discussion | Design discussion groups, focus groups |
| **11** | Mind maps | Graffiti walls, storyboards, intentional billboards, collages, photo studies |
| **12** | Evaluation | Heuristic assessment, evaluative research, rapid iterative testing and assessment, cognitive process browsing |
| **13** | Observation | Observation, Participant Observation, Covert Observation, Shadowing, Behavioral Maps |
| **14** | Prototype | Prototyping, parallel prototyping, empirical prototyping, elastic modeling |
| **15** | Participatory Design | Participatory Design, Participatory Action Research, Derivative Research |
| **16** | Role simulation | Role analysis, role play, body storming |
| **17** | Interview | Questionnaires, Interviews, Surveys, Triangulation, Triangulation |
| **18** | Case Studies | Design case studies, research by design |
| **19** | Scenario Analysis | Scenario Describing Swimming Lane Charts, Scenario Approach |
| **20** | Simulation | Simulation exercises, behind-the-scenes simulations |
| **21** | Stakeholder Studies | Stakeholder Analysis Chart, Stakeholder Browsing |
| **22** | Data Interpretation | Weighted Matrix,Semantic Difference Method,Likert Scale,SUS Scale |
