# Supplementary material for: How to choose a design thinking method for teaching the design of localization: A two-dimension linguistic fuzzy model with two-tuples
Source: PLoS One. 2024 May 30;19(5):e0300505. doi: 10.1371/journal.pone.0300505 (PMC11139305; doi:10.1371/journal.pone.0300505)
Supplement: Attachment — Submitted filename: Response to Reviewers.docx [file pone.0300505.s002.docx]

**Dear Editors and Reviewers:**

Thank you for your letter and for the reviewers’ comments concerning our manuscript entitled “How to choose a design thinking method for teaching the design of localization: A two-dimension linguistic fuzzy model with two-tuples” (ID: PONE-D-23-35366). Those comments are all valuable and very helpful for revising and improving our paper, as well as the important guiding significance to our researches.

We have carefully studied these comments and made major revisions which we hope will be approved. Revised portion are marked in red in the paper. The main corrections in the paper and the responds to the reviewer’s comments are as flowing. First, we modified the style of the paper according to the journal’s requirements, including those for file naming. Second, we uploaded the expert-assessed data as supporting information files, and upload the data to the figshare repository, https://doi.org/10.6084/m9.figshare.25010591. Third, we use the following measures to address the copyright issues of Figures 11 and 12:

1, The map of Fig. 11 is removed and the schematic map of the plane is redrawn. In addition, since the street view map was derived from the researcher's photographs, the street view source information was added to the caption of Figure 11.

2, Remove the map from Figure 12.

Finally, we responded to the reviewers' comments and submitted three revised manuscript documents: The first document is a revised version of the original manuscript (track changes), which does not have English proofreading; the second file is a revised manuscript of English proofreading; and the third file is a revised manuscript without trace version, which includes figures and tables. Reviewers can view the details of the revisions.

**Note:** If you need to verify the proofreading of a manuscript, please send an e-mail to contact@englishproofread.com with the subject line "Verification of Manuscript Authenticity Required" and the unique reference number 20240116_YLF_9591.

**Responds to the reviewer’s comments:**

------------------------------------------------------

We are very grateful for the reviewers' comments and will respond to each question in the "Reviewer Questions and Responses" section.

------------------------------------------------------

**Reviewer 1:**

(1) The problem of improving the clarity of language and grammar.

**Response:** We are very grateful to your comments for the manuscript. According to your advice, We have sent the article to a professional organization for English proofreading and have provided supporting documents.

(2) Recommendations for expanding the literature review.

**Response:** We appreciate and accept the reviewer's suggestions and have included "1.2 Locality and Design Teaching" and "1.2 Locality and Design Teaching" in the supplement some of the literature. In addition, "1.3 Selection of Teaching Methods" has been added to clarify the novelty of this study and to differentiate it from other studies in the field. For details, please refer to "1 Introduction".

Supplementary Literature:

Wu, Y. J., & Chen, J. C. (2021). Stimulating innovation with an innovative curriculum: a curriculum design for a course on new product development. The International Journal of Management Education, 19 (3), 100561.

Béchard, J. P., & Grégoire, D. (2005). Entrepreneurship education research revisited: The case of higher education. Academy of management learning & education, 4(1), 22-43.

Nabi, G., Liñán, F., Fayolle, A., Krueger, N., & Walmsley, A. (2017). The impact of entrepreneurship education in higher education: A systematic review and research agenda. Academy of management learning & education, 16(2), 277-299.

Manimala, M. J., & Thomas, P. (2017). Entrepreneurship education: innovations and best practices. Entrepreneurship education: Experiments with curriculum, pedagogy and target groups, 3-53.

Rasiah, R., Somasundram, S., & Tee, K. P. (2019). Entrepreneurship in education: Innovations in higher education to promote experiential learning and develop future ready entrepreneurial graduates. development, 6, 7.

Tan, T. A. G., & Vicente, A. J. (2019). An innovative experiential and collaborative learning approach to an undergraduate marketing management course: A case of the Philippines. The International Journal of Management Education, 17(3), 100309.

Thijs, A., & Van Den Akker, J. (2009). Curriculum in development. Netherlands Institute for Curriculum Development (SLO).

Powers, L. M., & Summers, J. D. (2009). Integrating graduate design coaches in undergraduate design project teams. International Journal of Mechanical Engineering Education, 37(1), 3-20.

Bernstein, W. Z., Ramanujan, D., Zhao, F., Ramani, K., & Cox, M. F. (2012). Teaching design for environment through critique within a project-based product design course. International Journal of Engineering Education, 28(4), 799.

De Vere, I., Melles, G., & Kapoor, A. (2010). Product design engineering–a global education trend in multidisciplinary training for creative product design. European journal of engineering education, 35(1), 33-43.

Eggink, W. (2009). A practical approach to teaching abstract product design issues. Journal of engineering design, 20(5), 511-521.

Samuel, A. B., & Rahman, M. M. (2018). Innovative teaching methods and entrepreneurship education: A review of literature. Journal of Research in Business, Economics and Management, 10(1), 1807-1813.

Goodyear, P. (2015). Teaching as design. Herdsa review of higher education, 2(2), 27-50.

Marjanovic, O. (2016). Designing innovative education through action design research: Method and application for teaching design activities in large lecture environments. Journal of Information Technology Theory and Application (JITTA), 17(2), 1.

Cameron, L. (2017). How learning designs, teaching methods and activities differ by discipline in Australian universities. Journal of learning design, 10, 69-84.

(3) Clearer articulation of the unique contributions and potential impacts of the research.

**Response:** Thanks to the reviewers' approval and comments on this study, we found by analyzing the past studies that the current methods of assessing the applicability of teaching methods from the literature analysis to the statistical analysis are the results of one dimension, and do not take the thematic characteristics of the course as the second dimension. and therefore the assessment of suitability is not comprehensive enough. For this reason, this study proposes a 2-dimensional expert assessment method, setting up two concepts of thematic character of the course and pedagogical mode, and adopting a 2-dimension linguistic fuzzy model with 2-tuples to establish mixed statistical methods, including Delphi method, triangular fuzzy number method, Euclidean distance method, and 2DLL method, 2DLWAA method.

We add the unique contributions and potential impacts of the research described above to "1.3 Selection of Teaching Methods" in the article.

(4) Provide a broader context for the impact of research.

**Response:** Through the literature review and actual teaching experience, it was found that design courses contain 2-dimensional attributes: first, the subject matter of the course, in addition to the "locality" subject matter described in this study, there may be other types of subject matter, such as intelligence, entrepreneurship, and marketing, etc. Second, the teaching mode of the course, in addition to "design practice", there may be participatory exploration, discussion, and experimentation. The second is the pedagogical mode of the course, which may include participation in exploration, discussion, and experimentation in addition to "design practice". In addition, the linguistic fuzzy model with 2-tuples is used to find the appropriate design thinking method, which makes the assessment process more comprehensive and avoids the problem of information loss. The assessment method proposed in this study requires experts to have enough experience in design teaching as well as a more comprehensive understanding of the types and application characteristics of design thinking methods, but there are some problems in the research process, such as the lack of application experience of certain experts in some infrequently used methods, and therefore the lack of sufficient judgment of the potential of the future application of such methods, which leads to the results of the affected assessment, for example, "Stakeholder studies" and "Data Interpretation". Therefore, this assessment method is advantageous for the integration and transformation of existing design thinking methods, while it has limitations for the innovation and development of thinking methods.

We add the implications of the research described above in "5 Conclusions".

------------------------------------------------------

**Reviewer 2:**

1. Some spelling and punctuation error issues in the article.

**Response:** We seriously thank the reviewers for their work and comments. According to your advice, We have sent the article to a professional organization for English proofreading and have provided supporting documents.
