# Supplementary material for: How to choose a design thinking method for teaching the design of localization: A two-dimension linguistic fuzzy model with two-tuples
Source: PLoS One. 2024 May 30;19(5):e0300505. doi: 10.1371/journal.pone.0300505 (PMC11139305; doi:10.1371/journal.pone.0300505)
Supplement: Attachment — Submitted filename: Response to Reviewers.doc [file pone.0300505.s003.doc]

**Dear Editors and Reviewers:**

Thank you for your letter and for the reviewers’ comments concerning our manuscript entitled “How to choose a design thinking method for teaching the design of localization: A two-dimension linguistic fuzzy model with two-tuples” (ID: PONE-D-23-35366R1). Those comments are all valuable and very helpful for revising and improving our paper, as well as the important guiding significance to our researches.

We have carefully studied these comments and made minor revisions which we hope will be approved. Revised portion are marked in red in the paper. The main corrections in the paper and the responds to the reviewer’s comments are as flowing.

**Responds to the reviewer’s comments:**

------------------------------------------------------

**Reviewer 1:**

1. The authors have clearly made efforts to improve the article, but there are still ambiguities regarding how expert knowledge was identified. It has not been sufficiently explained how exactly this knowledge was identified, which can be problematic for readers trying to understand the research process.

**Response:** We accept the reviewer's professional comments and add the following to "3.1 Summary of the design thinking method" and “3.2.2 Results of the evaluation of the applicability of teaching” of the manuscript:

In this study, expert knowledge was utilized to assess the applicability of various design thinking methods in university education, and there were two stages in the process. In the first stage, to select a method that matches the curriculum of "locality" thematic design, a group of five teachers was organized to form a panel of experts. First, the leader of the focus group made a preliminary summary of more than 100 design thinking methods and organized them into a visual chart according to the six phases of design teaching (Fig 6), and then the leader of the group explained to the other members the characteristics of the methods as well as analyzed the process of their application, and then asked each member to express their understanding and opinion about each design thinking method, and finally all members discussed the advantages and disadvantages of each method and selected 71 methods suitable for the design course, which were summarized into 22 categories.

Fig 6. Visualization charts for method selection (a small part).

In the second stage, to analyze the applicability of the selected 22 categories of methods in the dimensions of "design practice" and "locality", an evaluation team consisting of 36 experts was formed to evaluate the methods by the Delphi method, and the specific steps were as follows:

1, The team leader described and explained the characteristics and application process of the 22 methods to the members.

2, The opinions of the members were collected using questionnaire statistics, and each member could view the evaluation opinions of other members and encourage mutual discussion.

3, The questionnaires were all returned and valid, and finally, the assessment values of each member were integrated and analyzed.

The above will be added to the manuscript.

2. At the same time, it is worth considering whether uncertainty could have been modeled in a different way, perhaps more suitable for the analyzed problem. The authors should consider whether there are other methods of identifying expert knowledge that could have been used in this context, and whether the methodology employed, such as the RANCOM method, could have been more appropriate or simply more accessible to readers.

**Response:** Thanks to the reviewers' suggestions, we will add the following to "1.3 Selection of teaching methods" in the manuscript.

Evaluation information in the form of language is not directly involved in mathematical operations, and decision-making on it requires multiple experts to form a group decision, which then requires information transformation and aggregation of the information of each program, followed by sorting, comparison, and selection of the best. At present, the methods of processing linguistic evaluation information are mainly divided into three categories: one is the analytical method of evaluation information transformation, which transforms linguistic evaluation information into fuzzy numbers, and then carries out arithmetic operations and analysis, such as the fuzzy analytic hierarchy process (F-AHP) (Afolayan et al., 2020); the second is the symbol transfer-based analysis method, which is based on the calculation of linguistic evaluation information values directly from the set of linguistic evaluation information, such as RANking COMparison (RANCOM) (Wieckowski et al., 2023); the third category of methods is the 2-tuples linguistic analysis method (Malhotra & Gupta, 2023 ), which aims to transform the linguistic evaluation information given by the decision maker into 2-tuples linguistic symbols, and then use the 2-tuples linguistic algorithm and the agglomerative operator to perform information agglomerative analysis. By comparing and analyzing the above methods, it is found that the 2-tuples linguistic analysis method has great superiority in dealing with model and information loss, which makes the calculation results of linguistic evaluation information more accurate.

The following literature is added to the paper:

29.Wieckowski J, Kizielewicz B, Shekhovtsov A, Sałabun W. Rancom: A novel approach to identifying criteria relevance based on inaccuracy expert judgments. Engineering Applications of Artificial Intelligence. 2023; 122: 106114.

30.Afolayan AH, Ojokoh BA, Adetunmbi AO. Performance analysis of fuzzy analytic hierarchy process multi-criteria decision support models for contractor selection. Scientific African. 2020; 9: e00471.

31.Malhotra T, Gupta A. A systematic review of developments in the 2-tuple linguistic model and its applications in decision analysis. Soft Computing. 2023; 27(4): 1871-1905.

3. Furthermore, there is potential to increase attention to the sensitivity associated with the applied uncertainty generalization. The authors should consider the possibility of more detailed discussion of this issue to provide readers with a fuller understanding of the analyzed results.

**Response:** Thanks to the reviewer's suggestion, we will add the following content in "4.1 Selection of design thinking approaches in localized design courses" of the manuscript.

The actual assessment and decision-making problems are generally complex things, and the multi-criteria assessment, although it can synthesize multiple factors to analyze the problem, is still one-dimensional, and the mutual exclusion and influence between the criteria belongs to the continuous multi-dimensional structure. In addition, the ambiguity and uncertainty of human thinking, as well as the finite nature of individual knowledge, experience, and ability to solve the decision-making problems faced often do not have all the necessary knowledge and information if they rely only on individual experience and wisdom. Although groups of experts have certain common behavioral and professional characteristics at a particular time, such as cultural level and value orientation. However, the fact that these characteristics of expert groups are often abstract, the form of evaluation given to alternatives is uncertain linguistic information, and the difficulty for individuals to understand the full picture of the group and the sometimes one-sided understanding of the decision-making goals makes it difficult for individual decision-makers to accurately grasp the behavioral characteristics of the group. In this study, thematicity and practicability were two important dimensions in determining the appropriateness of the pedagogical methods used in the design course, and the two were not isolated, but rather in a state of interaction, which added to the complexity of the decision. When assessing the thematic dimension also affects the practicality, therefore, the method proposed in this study is a continuous linguistic representation of the evaluation information, which is more suitable for the 2 dimensions and avoids missing and distorted information. The preferred approach through visualization of graphs is more intuitive and precise than numerical. The main objective is to screen and develop appropriate design teaching methods by collecting group opinions from university teachers with rich design teaching experience, analyzing and evaluating them, and identifying the knowledge of the expert group.
